# Differences in Somatic Mutation Profiles between Korean Gastric Cancer and Gastric Adenoma Patients

**DOI:** 10.3390/jcm10092038

**Published:** 2021-05-10

**Authors:** Seung Woo Lee, Taekyu Lee, Hae Jung Sul, Ki Cheol Park, Joonhong Park

**Affiliations:** 1 Division of Gastroenterology, Department of Internal Medicine, College of Medicine, The Catholic University of Korea, Seoul 06591, Korea; leeseungw00@hanmail.net; 2 Thermo Fisher Scientific Solutions, Seoul 06349, Korea; taekyu.lee@thermofisher.com; 3 Department of Pathology, College of Medicine, The Catholic University of Korea, Seoul 06591, Korea; hjsul@cmcdj.or.kr; 4 Clinical Research Institute, Daejeon St. Mary’s Hospital, The Catholic University of Korea, Daejeon 34943, Korea; kcpark@cmcdj.or.kr; 5 Department of Laboratory Medicine, Jeonbuk National University Medical School and Hospital, Jeonju 54907, Korea; 6 Research Institute of Clinical Medicine of Jeonbuk National University-Biomedical Research Institute of Jeonbuk National University Hospital, Jeonju 54907, Korea

**Keywords:** mutation profiles, gastric adenoma, gastric cancer, mutant burden, next-generation sequencing

## Abstract

Background: We aimed to investigate molecular factors potentially related to the progression of gastric adenoma (GA) to gastric cancer (GC) and compare the mutation characteristics between GC and GA. Methods: We conducted custom gene panel sequencing for 135 GC-related genes and estimated the difference in somatic mutation profiles between 20 GC and 20 GA cases. Results: A total of 31 somatic mutations, including 22 missense, 3 nonsense, and 6 frameshift mutations, were detected in 17 samples. We estimated an average of 1.8 mutations per sample (range, 1 to 3 mutations), with 12 in GC and 5 in GA. GC tended to have one or more mutated genes (*p* = 0.0217), as well as higher allele frequencies of mutated genes (*p* = 0.0003), compared to GA. Likewise, known driver mutations associated with GC tumorigenesis (*TP53*, *ERBB2*, *PIK3CA*, and *RNF43*) were identified in half of the GC cases (50%, 10/20; *p* = 0.0002). Only the mutant burden, regardless of gene type, was retained, with an odds ratio of 1.8392 (95% confidence interval (CI), 1.0071 to 3.3588; *p* = 0.0474). Conclusion: Our study demonstrates that the accumulation of mutant burden contributes to tumorigenesis progression from GA to GC in Korean patients, regardless of the kind of genes. These findings may elucidate the molecular pathogenesis of gastric carcinogenesis and malignant progression.

## 1. Introduction

Gastric adenoma (GA) is a precancerous lesion that is a direct precursor to gastric adenocarcinoma, a type of GC [1]. Up to 50% of GA cases progress to gastric cancer (GC) [2], indicating the need for therapeutic removal of GAs, as well as the importance of elucidating progression mechanisms. GC shows a high mortality rate and incidence, particularly in East Asians, and approximately 90% of GCs are adenocarcinomas. Etiological and histological heterogeneity and ethnic differences complicate the genetic subtyping of GC, thus making it difficult to decide on standard treatment modalities and molecular classification systems [3]. A previous study adopting synchronous GA/GC pairs indicated that the emergence of histopathologically distinct subclones or the divergence of GA/GC pairs occurs early in gastric tumorigenesis [1]. Because of the loss of GA tissues during malignant progression, it is challenging to assess premalignant and malignant lesions in the stomach together, but residual GAs often continue to exist along with GC lesions. Similarly, GC in hyperplastic polyps follows a multistep progression model, a sequence from hyperplasia–dysplasia to adenocarcinoma [4]. However, it remains unknown as to whether GA includes sporadic genetic changes, and the molecular mechanisms of GA-to-GC progression remain unclear. Next-generation sequencing (NGS), which allows for the interrogation of numerous variants from multiple genes within a given tumor sample, has facilitated advances in cancer evolution studies [5,6,7]. GA-to-GC transition is encrypted within the genome and is an evolutionary process; therefore, evolutionary perspectives on genome-wide mutational distribution and abundance may demonstrate valuable insights into GC development, with potential clinical benefits.

In this study, we aimed to investigate molecular factors potentially associated with GA-to-GC progression by comparing the mutation characteristics between GC and GA using custom gene panel sequencing.

## 2. Materials and Methods

### 2.1. Study Samples

For the sample size of this study, we referred to previous studies that investigated somatic mutations in 20 and 34 patients with GC using NGS [8,9]. Similarly to these studies, we selected 20 cases of GC and compared them with 20 cases of GA. A total of 40 formalin-fixed, paraffin-embedded (FFPE) samples of gastric tissues were obtained from 40 Korean patients diagnosed with GC (advanced = 10, early = 10) or GA classified as intestinal type only (high-grade dysplasia (HGD) = 10, low-grade dysplasia (LGD) = 10) at Daejeon St. Mary’s Hospital (Daejeon, Korea). All the GC samples were obtained after surgical resection, and all the GA samples were obtained after endoscopic resection. We applied the following diagnostic criteria to classify the cancer staging and histological findings: the American Joint Committee on Cancer for cancer staging [10] and the World Health Organization (WHO) classification of tumors of the digestive system for histological findings [11]. Histological classifications from the same tissues were confirmed by a board-certified pathologist. FFPE samples that had more than 50% tumor content to be estimated were sectioned at a thickness of 10 micrometer and preserved in 1.5 mL microtubes. The blade was replaced to prevent the contamination of DNA for every tissue block.

### 2.2. DNA Isolation and Quantification

Genomic DNA was isolated from four or five unstained FFPE sections per sample using the RecoverAll Total Nucleic Acid Isolation Kit (Thermo Fisher Scientific, Waltham, MA, USA) according to the manufacturer’s instructions. The genomic DNA was assessed quantitatively using a Qubit 2.0 Fluorometer with a Qubit dsDNA High Sensitivity Assay Kit and a TaqMan RNase P Detection Reagent Kit (Thermo Fisher Scientific), and it was considered adequate when the DNA concentration was >10 ng/μL.

### 2.3. Custom Panel Design and Library Preparation

A custom panel targeting genes associated with GC or GA identified by previous research was designed using the Ion AmpliSeq Designer online tool (www.ampliseq.com, accessed on 17 April 2020). Targeted genes were chosen according to their reported associations with gastric cancer in published molecular studies (Supplementary Table S1) [5,6,8,9,12]. Libraries were prepared using Ion AmpliSeq Oncomine Research Panel primer pools and the Ion AmpliSeq Library Kit 2.0 Plus (Thermo Fisher Scientific) following the manufacturer’s recommendation. Briefly, 20 ng samples of genomic DNA isolates from two target amplification reactions were combined. The libraries for the custom panel were digested partially and phosphorylated using the FuPa reagent, ligated to different barcode adapters using the Ion Xpress Barcode Adapters 1–48 Kit (Thermo Fisher Scientific), and purified. The purified libraries were quantified using the Ion Library TaqMan Quantitation Kit (Thermo Fisher Scientific).

### 2.4. Sequencing Analysis Using the Ion S5XL

Pooled purified libraries of seven multiplexed tumor DNAs per chip at a concentration of 50 pM were loaded onto chips and analyzed using the Ion Chef with the Ion 540 chef Kit (Thermo Fisher Scientific) and sequenced on S5XL using Ion S540 chips (Thermo Fisher Scientific) for 200 base-read single-strand sequencing as per the manufacturer’s instructions.

### 2.5. Integrative Bioinformatic Analyses

Automated analyses of raw sequencing data were performed sequentially in the Torrent Suite software 5.10 using default analysis parameters. Data analyses for variant calling were performed using Ion Reporter 5.10 with Oncomine Knowledgebase Reporter (https://ionreporter.thermofisher.com/ir/, accessed on 23 August 2020 and the default settings. Briefly, the criterion of variant allele frequency for the preconfigured Torrent Variant Caller used the following parameters: minimum allele frequency (cutoff for supporting a variant) of InDel 0.08 and SNV 0.05; minimum coverage (total required for reads or no-call) of InDel 15 and SNV 15; and strand bias (proportion of variant alleles overwhelmingly from one strand) of InDel 0.9 and SNV 0.96. Most tumor samples were within the standards of sequencing results: mapped reads >2,000,000, uniformity >85%, on-target rate >85%, and mean depth >500×. Results with suspected errors and poor quality were excluded according to <100× coverage, <5% variant allele frequency, and variants in the out-of-coding region [13]. The final analysis of each case was reviewed and reported by a medical laboratory doctor. All genetic alterations were reported following standards and guidelines for interpreting sequence variants in cancer [14].

### 2.6. Statistical Analysis

Descriptive statistics were used to demonstrate the mean ± SD of the age and mutant burden of patients. Chi-square or Fisher’s exact tests were used to compare genetic differences between GC and GA according to the pathophysiological and/or histological findings. Normality was assessed using the Shapiro–Wilk test, and Student’s *t*-test was used to compare the means of mutant burdens between GC and GA. Multivariate logistic regression analysis was conducted to investigate independent factors related with GA-to-GC progression. Nonsignificant predictors were removed using the enter method (probability threshold for removal: 0.1). The diagnostic performance of factors for identifying patients with GC was assessed using the area under the receiver operating characteristic (ROC) curve (AUC). Statistical analysis was performed using MedCalc Statistical Software Version 19.5.3 (MedCalc Software Ltd., Ostend, Belgium). Two-tailed *p* < 0.05 was regarded to indicate statistical significance.

## 3. Results

### 3.1. Clinicopathologic Difference between Gastric Cancer and Adenoma

Upon comparing the clinicopathologic findings between GC and GA, intestinal metaplasia was found more frequently in GA than in GC (*p* = 0.0409). The clinicopathologic findings of 40 Korean patients with gastric cancer or adenoma are shown in Table 1.

### 3.2. Quality Control Metrics of Raw Sequencing Data

In quality control (QC) metrics for raw sequencing data generated from six independent experiments, the mean number of total usable reads was 31,187,553 (63%) and the mean read length was 104 bp (SD, 8; range, 95–122). The mean mapped read count, on-target read rate, mean depth of on-target regions, and uniformity were 5,423,026 bp, 95%, 898×, and 91.7%, respectively. All experiments satisfied the manufacturer’s specifications (>95% of amplicons should have a read depth of >500×).

### 3.3. Somatic Mutation Profiles

**Figure 1:**
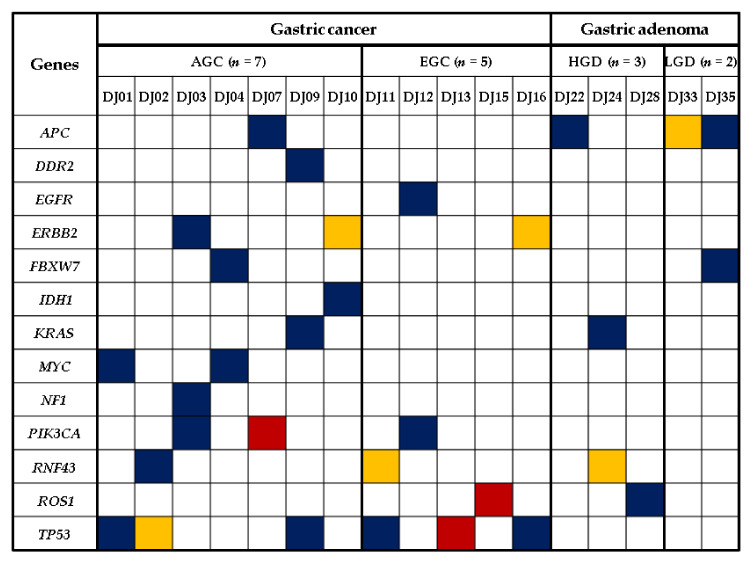
Distribution of somatic mutation profiles based on disease subtypes in 17 patients with gastric cancer or adenoma. Genes are indicated on the far left column, and each patient (DJOO) is depicted on the third row. AGC, advanced gastric cancer; EGC, early gastric cancer; HGD, gastric adenoma with high-grade dysplasia; LGD, gastric adenoma with low-grade dysplasia. Indigo, missense mutation; red, nonsense mutation; yellow, frameshift mutation.

A total of 4178 unfiltered variants were identified from the raw sequencing data using the Ion AmpliSeq custom panel. After variant filtering of cancer genes to determine potential genes of interest, 31 somatic SNVs or indels passed the data analysis algorithms. Among the 31 somatic mutations, 22 missense, 3 nonsense, and 6 frameshift mutations were detected in the 135 GC-related genes. Details of the somatic mutation profiles in 17 Korean patients with GC or GA are summarized in Table 2. We detected an average of 1.8 mutations per sample (range, 1 to 3 mutations) in 12 GC and 5 GA cases. The five most commonly mutated genes were *TP53* (*n* = 6), *APC* (*n* = 4), *ERBB2* (*n* = 3), *PIK3CA* (*n* = 3), and *RNF43* (*n* = 3). *TP53* was the most commonly mutated gene, with mutations found in three AGC and three EGC. The *APC* mutation was the second most frequent, found in one GC and three GA. The third most commonly mutated genes were *ERBB2*, *PIK3CA*, and *RNF43*, which were shared in seven GC and one GA. Collectively, 11 of the 12 GC cases with any mutations had somatic mutations in *TP53*, *ERBB2*, *PIK3CA*, or *RNF43* (Figure 1).

### 3.4. Genetic Differences between Gastric Cancer and Adenoma

**Figure 2:**
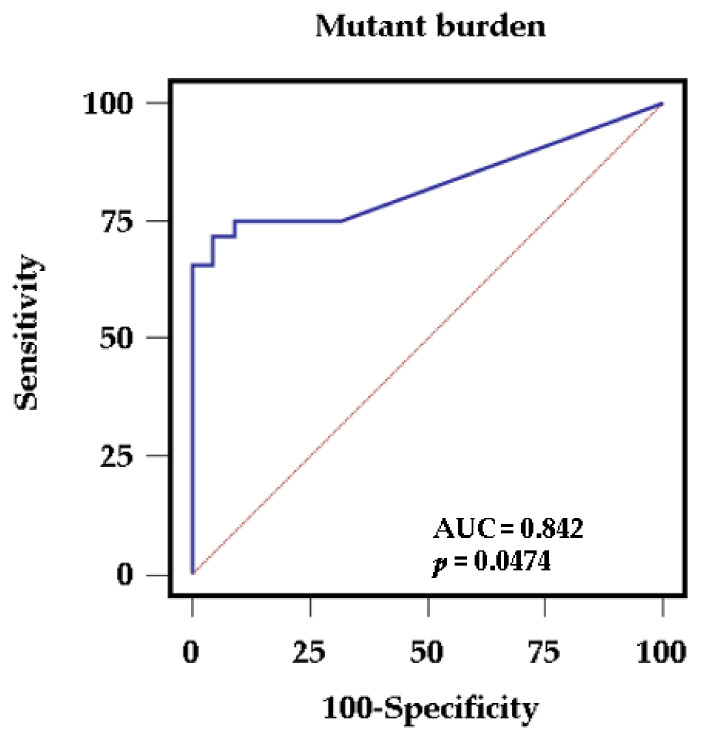
Receiver operating characteristic (ROC) curve for mutant burden for genetic differences between gastric cancer and gastric adenoma. The area under the curve (AUC) for mutant burden for the progression of gastric adenoma to gastric cancer is 0.842 with sensitivity of 72% and specificity of 95% (*p* = 0.0474).

Because the histopathology of GC differs from that of GA, genetic differences between GC and GA were investigated. GC had one or more mutated genes (*p* = 0.0217), as well as a higher allele frequency of mutated genes (*p* = 0.0003), compared to GA. Likewise, for GC, known driver mutations associated with GC tumorigenesis (*TP53*, *ERBB2*, *PIK3CA*, or *RNF43*) were identified in half of the GC samples (50%, 10/20; *p* = 0.0002) (Table 3). In an enter stepwise logistic regression model, only the mutant burden, regardless of the kind of gene, was retained with an odds ratio of 1.8392 (95% confidence interval (CI), 1.0071 to 3.3588; *p* = 0.0474). The ROC curve analysis for allele frequency of mutated genes showed significant diagnostic utility with an AUC of 0.842 (95% CI, 0.718 to 0.927) for GA-to-GC progression (Figure 2).

## 4. Discussion

GA is defined as localized polypoid proliferation of dysplastic epithelium of the stomach considered to represent neoplastic lesions with malignant potential, and endoscopic submucosal dissection (ESD) is the standard management for GA. The clinical signs of GA are not specific. It usually found incidentally during screening endoscopy. The WHO defines gastric adenoma as the presence of histologic unequivocal neoplastic epithelium without evidence of tissue invasion [11]. The subtypes of GA, based on their epithelial phenotypes, are intestinal type, gastric pyloric gland, and foveolar type adenoma. However, only GA classified as intestinal type was included in our study. Gastric dysplasia is divided into LGD and HGD on the basis of the degree of architectural distortion, cytoplasmic differentiation, mitotic activity, and nuclear atypia. The rate of malignant change ranges up to 23% for LGD and from 60 to 85% for HGD. The risks of metachronous HGD and metachronous gastric neoplasm (MGN) or GC after ESD did not differ between patients with LGD and those with HGD, despite the high risk reported in the HGD group. Otherwise, the HGD group showed substantially increased risk of GC or metachronous HGD compared to the LGD group in patients with no H. pylori infection [15]. The progression intervals are 4 to 48 months for HGD and 10 to 48 months for LGD [13]. GA with an absence of polarity of proliferating cells may favor carcinoma development, and tumor-associated macrophage number may be an independent risk factor, suggesting carcinoma development regardless of the follow-up duration [16]. Key molecular events may occur during early malignant transformation and may be recorded in somatic mutation profiles from GA-to-GC progression. The current management of dysplasia is ESD according to the recent guidelines [17,18,19]. The life expectancy of GC depends on the stage at the time of diagnosis. The 5-year survival rate of early GC is excellent at more than 90%; on the other hand, that of advanced GC with metastatic disease is less than 30% [20]. First-line standard treatment includes platinum compounds and fluoropyrimidines with trastuzumab for patients with *HER2*-positive GC [21]. Several alternative therapies for recurrent GC are available, such as ramucirumab, a monoclonal therapeutic antibody that inhibits *VEGF*-mediated tumor angiogenesis by binding with *VEGFR2*, alone or in combination with other cancer drugs [22]. However, 30 to 80% of the patients respond to treatment with ramucirumab or its combinations [23], suggesting that pharmacotherapy personalization is required to improve the efficacy of drug treatment.

In this study, we performed cancer gene panel sequencing to estimate differences in somatic mutation profiles in Korean patients with GC and GA. As a result, the number of mutated genes and mutant burden were increased in GC compared to GA. Consistent with the previous studies [1,24,25], the well-established somatic mutations *TP53*, *APC*, *ERBB2*, *PIK3CA*, *RNF43*, *FBWX7*, *KRAS*, *MYC*, and *ROS1* were recurrently detected in GAs or GCs in our study, suggesting that these mutations may contribute to potential drivers of early gastric tumorigenesis. Comparing the mutations detected in our nonsynchronous GAs with GCs with those in nonsynchronous GAs [26,27], half of the GC cases had exclusively somatic mutations associated with GC tumorigenesis identified in genes such as *TP53*, *ERBB2*, *PIK3CA*, or *RNF43*. However, only mutant burden, regardless of the kind of gene, was retained by logistic regression analysis. Similarly to our study, previous research did not identify any somatic changes in hyperplastic polyp components, even in genome-wide analyses comparing hyperplastic polyps and GA, in contrast to the adenocarcinoma components [7]. In a previous study on synchronous GA/GC pairs [1], most GA/GC pairs demonstrated parallel evolution with early divergence rather than stepwise evolution during GA-to-GC progression. Nonclonal synchronous GA/GC is frequent and the obtained GA genomes already have obvious genomic changes, suggesting that attention should be paid in the diagnosis of synchronous GA and GC, particularly in recurrent or residual cases. Genetic heterogeneity affects key tumorigenesis in cancer evolution, driving phenotypic variation, and a major cause of genetic heterogeneity in cancer is genomic instability. Genetic and epigenetic alterations, as well as altered tumor microenvironments, result in tumors made up of diverse subclones with different genetic and phenotypic characteristics. Diverse subclones can establish their cooperation through paracrine, cell–cell contact, and microenvironment remodeling, which allows them to exhibit a fitness advantage during tumor progression [28,29].

In a previous molecular analysis of GC [30], *TP53*-inactive and *TP53*-active GC included patients with intermediate recurrence rates and prognosis compared to the other two subtypes, and the *TP53*-active GC exhibited better prognosis. The oncogenic function of mutp53 is mainly caused by alterations in the structure and properties of mutp53 that allow binding with other oncogenic or tumor suppressive proteins [31]. Interestingly, five out of six GC cases with oncogenic *TP53* mutations such as p.Thr125Arg, p.Gln144His, p.His193Arg, p.Gly244Profs*16, and p.Glu286Lys in the DNA-binding domains had other somatic mutations of different genes, including *ERBB2*, *KRAS, MYC*, and *RNF43*. Consistent with frequent *TP53* mutation, elevated expression of *TP53* and aneuploidy were presented in the chromosomal instability (CIN) subtype [25]. Mouse models demonstrate that the genetic reconstitution of tumor suppression functions in the wild-type p53 rescues tumor growth. As promising therapeutic strategies, inhibitors of signaling pathways were modulated aberrantly by oncogenic mutant p53 proteins [32]. Somatic *PIK3CA* mutations have been reported in up to 25% of sporadic GCs and common molecular alterations in the Wnt and PI3K/Akt signaling pathways. The ratio of patients with high MSI was substantially lower, and EBV-positive and high-MSI characteristics seemed mutually inclusive in patients with GC [3,25]. We also found that there were three *PIK3CA* mutations in GC only. Inconsistent with the results of our study, gastric hyperplastic polyps with pyloric-type dysplasia were related to a *PIK3CA* mutation, whereas foveolar dysplasia carried *TP53* mutations [33]. Gene amplifications of *CCND1*, *CDK12*, *CCNE1*, and *ERBB2* were identified in patients with low tumor mutational burden [3]. We identified three *ERBB2* mutations representing missense or frameshift mutations that were identified in GC only. The amplification status of *ERBB2* and other genes should be investigated due to intratumoral heterogeneity of *ERBB2* amplification as a critical factor [34]. Particularly, the systemic therapeutic options for advanced GC have evolved rapidly to incorporate targeted molecular therapy with biomarker selection. In advanced GC overexpressing *HER2*, a combination of trastuzumab with platinum-based chemotherapy has become a standard treatment as a front-line therapy [35]. In addition, three *RNF43* mutations were identified in different histopathologic samples from AGC, EGC, and GA with HGD. *RNF43* mutation plays a key role as a tumorigenic driver from adenoma to carcinoma in early gastric carcinogenesis, and mutation in the tumor suppressor *RNF43* and dysregulated Wnt signaling are involved in multistep gastric carcinogenesis through the adenoma-to-carcinoma sequence [27]. Cells lacking the functional RNF43 protein do not react to either radiation treatment or chemotherapy, and they may also accumulate additional mutations that could further aggravate disease prognosis and outcomes. Thus, *RNF43* could serve as a significant GC biomarker to increase the ability to expect responses to adjuvant chemotherapy and thereby improve prognostic outcomes within the context of personalized medicine [36].

Contrary to colorectal adenocarcinoma, gastric adenocarcinoma predominantly arises from adenoma precursors and harbors truncating *APC* mutations, while only a minority of differentiated GCs contain *APC* mutations. This suggests that most gastric adenocarcinomas do not arise from adenoma precursors [37]. In our study, even though *APC* mutations were detected more frequently in GA (*n* = 3) than in GC (*n* = 1), the difference was not significant. *APC* mutations were mutually exclusive, which is consistent with their ability to activate Wnt–β-catenin signaling [38]. Similar to a previous study [27], we found that no GA or GC cases possessed both *APC* and *RNF43* mutations. The function of the *APC* gene in chromosomal stability and mitosis might disappear, and G1 might be arrested, with a high quantity of DNA in the S phase. Particularly, *APC* mutations alter cell cycle regulation and protein expression in the diffuse type of gastric adenocarcinoma [39].

Several limitations characterize this study. First, our samples were not matched pairs of GA and GC that originated from the same individual. Thus, intertumoral bias may have affected our ability to precisely investigate spatiotemporal genetic diversity. Contrary to our expectations, the numbers of putative mutations and copy number alterations were not significantly different between GC and GA. Recently, in paired continuous lesions representing gastric tumorigenesis, cancer-like changes occurred in low-grade intraepithelial neoplasia and accumulated in high-grade intraepithelial neoplasia and early GC during intestinal-type GC tumorigenesis. The signatures of five genes, *TIMP1*, *PLEKHS1*, *RGN*, *LAMP3*, and *GADD45B*, detected from the tumorigenesis process demonstrated robust prognostic significance for survival and relapse in GC patients, and this might result in the generation of potential molecular targets for the development of precision therapy [40]. Furthermore, transcriptome analysis using NGS may reveal potential molecular mechanisms underlying ramucirumab resistance and allow us to personalize the prescription of ramucirumab for GC [23]. Second, we did not assess MSI status associated with *TP53* or the presence of EBV infection related to *PIK3CA*. Future studies should investigate whether differences between GC and GA are affected by the occurrence of MSI or EBV infection. Third, the numbers of cases of each histopathological subtype were relatively small, and the patients were drawn from a single center, although each GC and GA subtype was well classified. Our results may therefore not be representative of the whole population of Korean GC and GA patients. Moreover, it is possible that no calls of mutations in the remaining 23 studied patients were due to a low tumor burden carrying “actual” mutations, even though FFPE samples had more than 50% tumor content to be estimated. Further molecular analysis is required to discover the mutational contribution to gastric tumorigenesis involving larger cohorts of samples with carefully curated clinically based data coupled with detailed histological data and complemented by functional analysis.

## 5. Conclusions

In conclusion, our study demonstrates that the accumulation of mutant burden contributes to the progression of tumorigenesis from GA to GC in Korean patients, regardless of the kind of genes. These findings may elucidate the molecular pathogenesis of gastric carcinogenesis and malignant progression. The discovery of diverse molecular characteristics by comprehensive NGS demonstrates more possibilities for both immunotherapies and targeted therapies of patients with GC arising from GA.

## Figures and Tables

**Table 1:** Clinicopathologic findings of 40 Korean patients with gastric cancer or gastric adenoma.

Findings	Total (*n =* 40)	Gastric Cancer (*n* = 20)		Gastric Adenoma (*n* = 20)		*p* Value
		10 AGC	10 EGC	10 HGD	10 LGD	
Male	27	5	8	7	7	0.7389
Age (Mean ± SD), year	70.9 ± 10.3	70.1 ± 13.9	69.7 ± 8.1	72.2 ± 7.9	71.7 ± 11.5	0.5353
Site						0.5346
Antrum	20	3	6	6	5	
Body	19	7	3	4	5	
Cardia	1	0	1	0	0	
Pathologic stage						N/A
I	10	0	10	N/A	N/A	
II	6	6	0	N/A	N/A	
III	3	3	0	N/A	N/A	
IV	1	1	0	N/A	N/A	
Differentiation						N/A
Well	0	0	0	N/A	N/A	
Moderately	11	4	7	N/A	N/A	
Poorly	9	6	3	N/A	N/A	
Lauren classification						N/A
Intestinal	6	4	2	N/A	N/A	
Diffuse	9	6	3	N/A	N/A	
Mixed	5	0	5	N/A	N/A	
Invasion site						N/A
Lymphatic	18	10	8	N/A	N/A	
Venous	13	9	4	N/A	N/A	
Perineural	6	5	1	N/A	N/A	
Subtype of adenoma						N/A
Intestinal	N/A	N/A	N/A	10	10	
Foveolar	N/A	N/A	N/A	0	0	
Gastric pyloric gland	N/A	N/A	N/A	0	0	
Intestinal metaplasia	28	4	7	10	7	0.0409
H. pylori infection	25	5	7	6	7	0.7471
Family history	3	1	0	1	1	0.5533
Alcohol	8	0	4	1	3	1.0000
Smoking	4	1	2	0	1	0.2980

AGC, advanced gastric cancer; EGC, early gastric cancer; HGD, gastric adenoma with high-grade dysplasia; LGD, gastric adenoma with low-grade dysplasia; N/A, not available.

**Table 2:** Details of somatic mutation profiles in 17 Korean patients with gastric cancer or gastric adenoma.

SN	Dx	Gene	Transcript	Base Change	AA Change	Effect	AF (%)	COSMIC ID
DJ01	AGC	*MYC*	NM_002467.6	c.404A > C	p.Asp135Ala	Missense	27.8	COSM9213747
DJ01	AGC	*TP53*	NM_000546.5	c.578A > G	p.His193Arg	Missense	37.7	COSM10742
DJ02	AGC	*RNF43*	NM_017763.6	c.367G > C	p.Ala123Prp	Missense	19.3	COSM4755837
DJ02	AGC	*TP53*	NM_000546.5	c.730_740del	p.Gly244Profs*16	Frameshift	31.3	N/A
DJ03	AGC	*NF1*	NM_000267.3	c.1264G > A	p.Ala422Thr	Missense	13.3	COSM3729095
DJ03	AGC	*ERBB2*	NM_004448.3	c.2263T > A	p.Leu755Met	Missense	29.2	COSM1205571
DJ03	AGC	*PIK3CA*	NM_006218.4	c.1035T > A	p.Asn345Lys	Missense	24.8	COSM754
DJ04	AGC	*FBXW7*	NM_0043303.6	c.1138G > A	p.Asp380Asn	Missense	6.5	COSM6197887
DJ04	AGC	*MYC*	NM_002467.6	c.221C > T	p.Pro74Leu	Missense	18.6	COSM1166663
DJ07	AGC	*APC*	NM_000038.6	c.775C > T	p.Arg259Trp	Missense	9.9	COSM2990952
DJ07	AGC	*PIK3CA*	NM_006218.4	c.1636C > T	p.Gln546*	Nonsense	23.2	COSM24712
DJ09	AGC	*DDR2*	NM_006182.4	c.398G > A	p.Arg133Gln	Missense	13.3	COSM6922479
DJ09	AGC	*KRAS*	NM_033360.4	c.35G > T	p.Gly12Val	Missense	21.2	COSM520
DJ09	AGC	*TP53*	NM_000546.5	c.374C > G	p.Thr125Arg	Missense	21.6	COSM45243
DJ10	AGC	*ERBB2*	NM_004448.3	c.2315_2320dup	p.Val773_Met774insAsnVal	Frameshift	33.4	COSM20959
DJ10	AGC	*IDH1*	NM_005896.3	c.367G > A	p.Gly123Arg	Missense	27.5	COSM96922
DJ11	EGC	*RNF43*	NM_017763.6	c.1977delT	p.Ser661Profs*39	Frameshift	12.0	COSM1734865
DJ11	EGC	*TP53*	NM_000546.5	c.856G > A	p.Glu286Lys	Missense	20.1	COSM10726
DJ12	EGC	*EGFR*	NM_005228.5	c.1474A > G	p.Ser492Gly	Missense	7.9	COSM236671
DJ12	EGC	*PIK3CA*	NM_006218.4	c.3127A > T	p.Met1043Leu	Missense	23.8	COSM5731063
DJ13	EGC	*TP53*	NM_000546.5	c.916C > T	p.Arg306*	Nonsense	11.4	COSM10663
DJ15	EGC	*ROS1*	NM_002944.2	c.5854G > T	p.Gly1952*	Nonsense	16.1	N/A
DJ16	EGC	*ERBB2*	NM_004448.3	c.2313_2314insCATTAC	p.Ala771_Tyr772insHisTyr	Frameshift	7.6	COSM20959
DJ16	EGC	*TP53*	NM_000546.5	c.432G > T	p.Gln144His	Missense	19.1	COSM45076
DJ22	HGD	*APC*	NM_000038.6	c.3358G > A	p.Gly1120Arg	Missense	11.6	COSM19329
DJ24	HGD	*KRAS*	NM_033360.4	c.351A > C	p.Lys117Asn	Missense	8.3	COSM19940
DJ24	HGD	*RNF43*	NM_017763.6	c.2228_2229del	p. Gly743Alafs*3	Frameshift	29.6	COSM8520865
DJ28	HGD	*ROS1*	NM_002944.2	c.6095G > C	p. Gly2032Ala	Missense	5.9	COSM6012441
DJ33	LGD	*APC*	NM_000038.6	c.1742delA	p.Lys581Argfs*9	Frameshift	7.5	COSM1432181
DJ35	LGD	*APC*	NM_000038.6	c.688C > T	p.Arg230Cys	Missense	7.1	COSM1696039
DJ35	LGD	*FBXW7*	NM_004958.4	c.1647T > A	p.Phe549Leu	Missense	6.9	COSM9232927

SN, sample number; Dx, diagnosis; AA, amino acid; AF, allele frequency; COSMIC ID, Catalogue of Somatic Mutations in Cancer Internal Database; AGC, advanced gastric cancer; EGC, early gastric cancer; HGD, gastric adenoma with high-grade dysplasia; LGD, gastric adenoma with low-grade dysplasia; N/A, not available.

**Table 3:** Somatic mutation profiles in 17 Korean patients with gastric cancer or gastric adenoma.

Findings	Total (*n =* 17)	Gastric Cancer (*n =* 20)		Gastric Adenoma (*n =* 20)		*p* Value
		10 AGC	10 EGC	10 HGD	10 LGD	
Mutated genes						0.0217
0	23	3	5	7	8	
1	5	0	2	2	1	
≥2	12	7	3	1	1	
Mutant burdens % (Mean ± SD)	17.9 ± 9.2	23.9 ± 7.8	14.8 ± 6.0	8.1 ± 2.6	7.2 ± 0.3	0.0003
Mutation type *						0.5294 *
Missense	22	13	4	3	2	
Nonsense	3	1	2	0	0	
Frameshift	6	2	2	1	1	
Recurrent mutations						0.0002 **
*APC*	4	1	0	1	2	
*ERBB2*	3	2	1	0	0	
*PIK3CA*	3	2	1	0	0	
*RNF43*	3	1	1	1	0	
*TP53*	6	3	3	0	0	

* Actual number of mutations was enumerated, but not number of patients. ** Recurrent mutations of *ERBB2*, *PIK3CA*, *RNF43*, and *TP53* except *APC* genes were compared. AGC, advanced gastric cancer; EGC, early gastric cancer; HGD, gastric adenoma with high-grade dysplasia; LGD, gastric adenoma with low-grade dysplasia; SD, standard deviation.

## Data Availability

The data presented in this study are available on request from the corresponding author.

## Supplementary Materials

The following are available online at https://www.mdpi.com/article/10.3390/jcm10092038/s1, Table S1: A list of 135 selected genes associated with gastric cancer or adenoma for next-generation panel sequencing.
