# Supplementary material for: Differences in Somatic Mutation Profiles between Korean Gastric Cancer and Gastric Adenoma Patients
Source: J Clin Med. 2021 May 10;10(9):2038. doi: 10.3390/jcm10092038 (PMC8126162; doi:10.3390/jcm10092038)
Supplement: Supplementary file 1 [file jcm-10-02038-s001.zip › jcm-1161634-supplementary.pdf]

Supplementary Table S1. A list of 135 selected genes associated with gastric cancer or adenoma for next-generation panel sequencing.

ABCB1	*CNGA4*	*GLI3*	*MET*	*RASA1*
ABL1	*CREBBP*	*GNAS*	*MLH1*	*RGNEF*
ABCA10	*CTNNA1*	*GNAQ*	*MSH6*	*RECQL*
ACVR2A	*CTNNA2*	*GSR*	*MTOR*	*RET*
ADRA1B	*CTNNB1*	*HSP90B1*	*MUC6*	*RHOA*
AKAP9	*CYP2D6*	*IDH1*	*MYC*	*RNF43*
AKAP13	*DDR2*	*ITGAV*	*MYH11*	*RNF213*
ALK	*DLC1*	*ITK*	*MYST4*	*ROS1*
APC	*DNAH7*	*JAK2*	*NSD1*	*RYR1*
ARHGAP5	*DNMT3A*	*JAK3*	*NRAS*	*SMAD2*
ARID1A	*DRD2*	*KAT6B*	*NF1*	*SMAD4*
ATM	*EGFR*	*KCNH6*	*NOTCH1*	*SMARCA4*
ATRX	*EIF2C4*	*KDM6A*	*NOTCH2*	*SMARCB1*
AURKA	*ELF3*	*KDR*	*NTRK1*	*SMO*
BCOR	*EP300*	*KIF2B*	*PCDH9*	*SOHLH2*
BNC2	*ERBB2*	*KIT*	*PDCD1LG2*	*STK11*
BRAF	*ERBB3*	*KMT2A*	*PIK3CA*	*SYNE1*
BRCA1	*EYA4*	*KMT2B*	*PIK3R1*	*TGFBR1*
BRCA2	*FAM46D*	*KMT2C*	*PKHD1*	*TGFBR2*
CAT	*FANCM*	*KMT2D*	*PLB1*	*TMPRSS2*
CCNE1	*FAT4*	*KRAS*	*PMS2*	*TOP1*
CD44	*FBXW7*	*LDOC1*	*POLR3A*	*TP53*
CD274	*FGFR1*	*LRP2*	*PRB2*	*TYR*
CDH1	*FGFR2*	*MACF1*	*PRKCB*	*VEGFA*
CDK6	*FGFR3*	*MAP2K1*	*PTCH1*	*ZAN*
CDK12	*FLT3*	*MAP2K4*	*PTEN*	*ZIC4*
CDKN2A	*GATA3*	*MDM2*	*PTPRC*	*ZNF217*
